# Functional differences between AMPK α1 and α2 subunits in osteogenesis, osteoblast-associated induction of osteoclastogenesis, and adipogenesis

**DOI:** 10.1038/srep32771

**Published:** 2016-09-07

**Authors:** Yu-gang Wang, Xiu-guo Han, Ying Yang, Han Qiao, Ke-rong Dai, Qi-ming Fan, Ting-ting Tang

**Affiliations:** 1 Shanghai Key Laboratory of Orthopedic Implants, Department of Orthopedic Surgery, Shanghai Ninth People’s Hospital, Shanghai JiaoTong University School of Medicine, 639 Zhizaoju Road, Shanghai 200011, People’s Republic of China

## Abstract

The endocrine role of the skeleton-which is impaired in human diseases including osteoporosis, obesity and diabetes-has been highlighted previously. In these diseases, the role of AMPK, a sensor and regulator of energy metabolism, is of biological and clinical importance. Since AMPK’s main catalytic subunit α has two isoforms, it is unclear whether functional differences between them exist in the skeletal system. The current study overexpressed AMPKα1 and α2 in MC3T3-E1 cells, primary osteoblasts and mouse BMSCs by lentiviral transduction. Cells overexpressing AMPKα2 showed higher osteogenesis potential than AMPKα1, wherein androgen receptor (AR) and osteoactivin played important roles. RANKL and M-CSF were secreted at lower levels from cells overexpressing α2 than α1, resulting in decreased osteoblast-associated osteoclastogenesis. Adipogenesis was inhibited to a greater degree in 3T3-L1 cells overexpressing α2 than α1, which was modulated by AR. An abnormal downregulation of AMPKα2 was observed in human BMSCs exhibiting the fibrous dysplasia (FD) phenotype. Overexpression of AMPKα2 in these cells rescued the defect in osteogenesis, suggesting that AMPKα2 plays a role in FD pathogenesis. These findings highlight functional differences between AMPKα1 and α2, and provide a basis for investigating the molecular mechanisms of diseases associated with impaired functioning of the skeletal system.

Several important hormones secreted by bone cells regulate energy balance and mineral ion homeostasis. This endocrine function of the skeleton is impaired in various diseases including osteoporosis, obesity, and diabetes-associated bone diseases^1^. Elucidating the molecular basis for the regulation of energy metabolism and hormone production in the skeleton is therefore of biological and clinical importance, and can provide insight into the pathogenesis of these diseases.

Adenosine triphosphate (ATP) is an immediate source of energy in living cells and must therefore be maintained at a relatively high level. In eukaryotic cells, the adenosine monophosphate (AMP)-activated protein kinase (AMPK) signaling cascade detects and initiates a response to decreases in cellular ATP concentration^2^ by coupling changes in the intracellular level of ATP to the phosphorylation of downstream substrates, resulting in increases or decreases in the rates of ATP production and consumption, respectively^3^.

Bone is a dynamic organ that is continuously remodeled throughout the lifetime of an organism and is susceptible to alterations in metabolic status and physiological state. Recent studies have revealed that bone metabolism is regulated by the brain and is closely linked to whole body energy homeostasis^1^,^4^,^5^. There are two main neuronal populations within the arcuate nucleus of the hypothalamus regulating energy homeostasis: The orexigenic, appetite-stimulating neurons and the anorexigenic, appetite-suppressing neurons. They encompass some of the most effective control of energy homeostasis in the entire body. In addition, they also are involved in regulating of skeletal homeostasis and linking the processes of bone and energy homeostasis. Remarkably, the number of central neuropeptides and neural factors regulating bone and energy homeostasis keeps growing. These neuronal pathways represent a growing area with intensive research interest that is looking for novel regulatory axes between the brain and the bone. As a sensor of energy metabolisms, the *in vitro* and *in vivo* evidence for AMPK regulation of osteoblast differentiation is controversial^6^,^7^,^8^, and the precise role of AMPK in bone metabolism remains an open question.

Mammalian AMPK comprises α, β, and γ subunits in a heterotrimeric complex^9^,^10^. The α subunit has two isoforms, α1 and α2, and contains a kinase domain at the N terminus, which is phosphorylated at Thr172 by upstream kinases^11^. The α1 subunit is widely expressed, whereas the α2 subunit is highly expressed in skeletal and cardiac muscle and in the liver^12^; a recent study showed that AMPK activation by electrical stimulation of rat hindlimb muscle involved the α2 isoform^13^. However, it is unclear whether the α1 and α2 isoforms have distinct biological functions in the skeletal system.

The present study investigated whether functional differences exist between AMPK α1 and α2 subunits with respect to osteogenesis, osteoblast-associated induction of osteoclastogenesis, and adipogenesis. The results indicated that the subunit composition of AMPK determines the susceptibility of MC3T3-E1, 3T3-L1, primary osteoblasts and bone marrow stromal cells (BMSCs) to osteogenic, osteoclastogenic, and adipogenic induction, which involved androgen receptor (AR), osteoactivin, macrophage colony-stimulating factor (M-CSF), and receptor activator of nuclear factor κB ligand (RANKL). Interestingly, an aberrant downregulation of the α2 subunit was associated with the fibrous dysplasia (FD) phenotype in BMSCs characterized by impaired osteogenesis, which was rescued by overexpressing the α2 subunit. These findings highlight functional differences between AMPKα1 and α2, and provide a basis for investigating the molecular mechanisms of diseases associated with impaired functioning of the skeletal system.

## Results

### AMPK α1 and α2 mRNA expression is upregulated during osteogenesis

**Figure 1:**
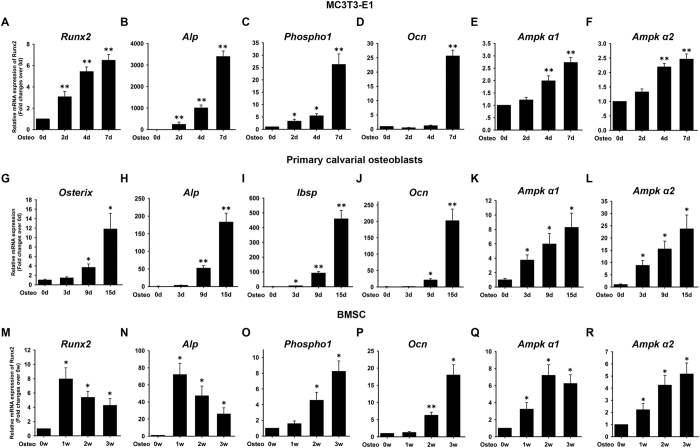
mRNA expression of AMPK α1 and α2 subunits during osteogenesis in MC3T3-E1, primary calvarial osteoblasts and mouse BMSCs. MC3T3-E1, primary osteoblasts and BMSCs were induced to osteogenic differentiation. The expression of *Runx2* (**A,M**), *Osterix* (**G**), *Alp* (**B,H,N**), *Phospho1* (**C,O**), *Ibsp* (**I**) *Ocn* (**D,J,P**), *Ampk α1* (**E,K,Q**) and *Ampk α2* (**F,L,R**) was evaluated by qRT-PCR. β-actin was used as internal control. The results are expressed as fold changes in mRNA abundance relative to 0d or 0w. Data are shown as mean ± SD. *P < 0.05; **P < 0.01, *vs.* 0d or 0w.

MC3T3-E1 cells used in the current study are preosteoblasts derived from mouse calvaria and have been used extensively as an *in vitro* model system to examine the osteogenic differentiation. On days 0, 2, 4, and 7 after induction, the expression of Runt-related transcription factor 2/core-binding factor α1 (*Runx2*), alkaline phosphatase (*Alp*), *Phospho1*, osteocalcin (*Ocn*), and *Ampk α1* and *α2* subunits was examined by qRT-PCR. An increase in the transcript levels of *Runx2* (Fig. 1A), *Alp* (Fig. 1B), and *Phospho1* (Fig. 1C) between days 2 and 7 and *Ocn* (Fig. 1D) on day 7 relative to day 0 was observed, indicating that MC3T3-E1 cells differentiated into osteoblasts. *Ampk α1* (Fig. 1E) and *α2* (Fig. 1F) mRNA expression was also upregulated on days 4 and 7 after induction.

Primary calvarial osteoblasts were induced to osteogenesis. On days 0, 3, 9, and 15 after induction, the expression of *Osterix, Alp, Ibsp, Ocn*, and *Ampk α1* and *α2* subunits was examined by qRT-PCR. An increase in the transcript levels of *Osterix* (Fig. 1G), *Alp* (Fig. 1H), *Ocn* (Fig. 1J) between days 9 and 15 and *Ibsp* (Fig. 1I) between days 3 and 15 relative to day 0 was observed. *Ampk α1* (Fig. 1K) and *α2* (Fig. 1L) mRNA expression was also upregulated between days 3 and 15 compared with day 0.

Also, BMSCs were induced to osteogenesis. On weeks 0, 1, 2, and 3 after induction, the expression of *Runx2, Alp, Phospho1, Ocn*, and *Ampk α1* and *α2* subunits was examined by qRT-PCR. As an early-stage marker gene of osteogenic differentiation and one of the most important transcriptional factors to initiate osteogenesis, *Runx2* mRNA levels showed a biphasic response during osteogenic differentiation, that is, increased from weeks 0 to 1 and reached its maximal levels at weeks 1 and thereupon fell progressively till weeks 3. Of these, there were statistically higher mRNA levels from weeks 1 to 3, relative to weeks 0 (Fig. 1M). An increase in the transcript levels of *Alp* (Fig. 1N) between weeks 1 and 3 and *Phospho1* (Fig. 1O) and *Ocn* (Fig. 1P) between weeks 2 and 3 relative to day 0 was observed, indicating that BMSCs differentiated into osteoblasts. *Ampk α1* (Fig. 1Q) and *α2* (Fig. 1R) mRNA expression was also upregulated between weeks 1 and 3 compared with week 0.

### Osteogenic potential is enhanced in cells overexpressing AMPK α2 compared with AMPK α1

**Figure 2:**
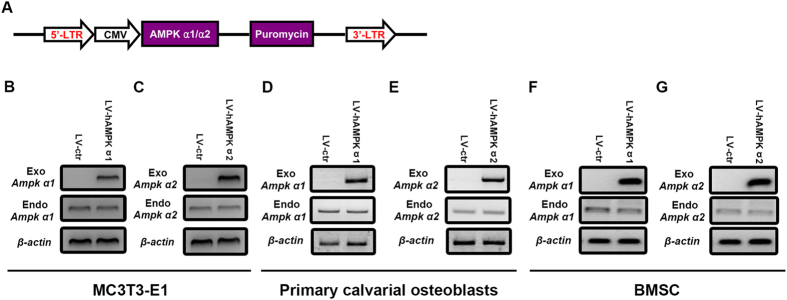
Generation of MC3T3-E1, primary osteoblasts and mouse BMSCs stably over-expressing AMPK α1 and α2 subunits. The AMPK α1 and α2 subunit coding sequences were cloned into an LV vector with a CMV promoter and positive clones were selected with puromycin (**A**). An empty LV vector served as a control (LV-ctr). RT-PCR was employed to check the expression of exogenous and endogenous α1 (**B,D,F**) and α2 (**C,E,G**) subunits of AMPK was evaluated. β-actin was used as an internal control.

To investigate whether there are functional differences between AMPK α1 and α2 subunits, exogenous AMPK α1 and α2 subunits were constitutively expressed in MC3T3-E1, primary osteoblasts and mouse BMSCs. The AMPK α1 and α2 subunit coding sequences were cloned into lentivirus (LV) vectors that were transduced into MC3T3-E1, primary osteoblasts and BMSCs (Fig. 2A), with the empty LV vector serving as a control. In MC3T3-E1, primary osteoblasts and BMSCs, exogenous AMPK α1 and α2 subunits were overexpressed in LV-AMPKα1 (Fig. 2B,D,F) and LV-AMPKα2 (Fig. 2C,E,G) clones respectively, as determined by RT-PCR. Moreover, the overexpression of exogenous AMPK α1 and α2 had no effect on the levels of the endogenous AMPK α1 and α2 (Fig. 2B–G).

**Figure 3:**
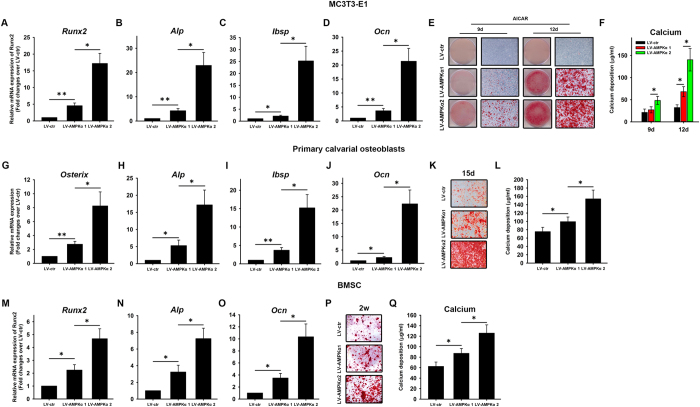
Osteogenesis in MC3T3-E1, primary osteoblasts and mouse BMSCs expressing AMPK α1 and α2. MC3T3-E1 cells were induced with osteogenic differentiation. On day 7 after induction, the expression of the osteogenesis markers *Runx2* (**A**), *Alp* (**B**), *Ibsp* (**C**), and *Ocn* (**D**) was evaluated by qRT-PCR. On days 9 and 12 after application of BMP2 and the AMPK agonist AICAR, calcium deposition was assessed by Alizarin Red staining (**E**) and quantification (**F**). Primary osteoblasts were induced with osteogenic differentiation. On day 9 after induction, the expression of *Osterix* (**G**), *Alp* (**H**), *Ibsp* (**I**) and *Ocn* (**J**) was evaluated by qRT-PCR. On days 15 after induction, calcium deposition was assessed by Alizarin Red staining (**K**) and quantification (**L**). Mouse BMSCs were induced with osteogenic differentiation. On day 7 after induction, the expression of *Runx2* (**M**), *Alp* (**N**), and *Ocn* (**O**) was evaluated by qRT-PCR. On days 14 after induction, calcium deposition was assessed by Alizarin Red staining (**P**) and quantification (**Q**). β-actin was used as an internal control of qRT-PCR. Data are shown as mean ± SD and are expressed as fold changes in mRNA abundance relative to LV-ctr cultures. *P < 0.05, **P < 0.01.

The AMPK α1- and α2-expressing MC3T3-E1 cells were induced to osteogenesis. On day 7 after induction, the expression of the osteogenesis markers *Runx2, Alp, Bsp*, and *Ocn* was assessed by qRT-PCR. Compared with LV-ctr, the α1- and α2-expressing cells had a significant upregulation in *Runx2, Alp, Ibsp* and *Ocn* (Fig. 3A–D). More importantly, a significant upregulation in *Runx2, Alp, Ibsp*, and *Ocn* transcript levels was detected in LV-AMPKα2 relative to LV-AMPKα1 cells (Fig. 3A–D). Consistent with these results, when the AMPK agonist AICAR was added in combination with BMP2, mineralization evaluated by Alizarin Red staining (Fig. 3E) and quantification (Fig. 3F) was greater in LV-AMPKα2 than in LV-AMPKα1 cells on days 9 and 12.

Primary osteoblasts overexpressing AMPK α1 or α2 subunits were induced to osteogenesis. On day 9 after induction, the α1- and α2-expressing primary osteoblasts had a significant upregulation in *Osterix, Alp, Ibsp* and *Ocn* (Fig. 3G–J) compared with LV-ctr. A significant upregulation in *Osterix, Alp, Ibsp* and *Ocn* transcript levels was detected in LV-AMPKα2 relative to LV-AMPKα1 cells (Fig. 3G–J). Consistent with these results, the Alizarin Red staining (Fig. 3K) and quantification (Fig. 3L) results revealed greater mineralization in LV-AMPKα2 than in LV-AMPKα1 cells on days 15.

BMSCs overexpressing AMPK α1 or α2 subunits were induced to osteogenesis. On day 7 after induction, the α1- and α2-expressing BMSCs had a significant upregulation in *Runx2, Alp*, and *Ocn* (Fig. 3M–O) compared with LV-ctr. A significant upregulation in *Runx2, Alp*, and *Ocn* transcript levels was detected in LV-AMPKα2 relative to LV-AMPKα1 cells (Fig. 3M–O). Consistent with these results, the Alizarin Red staining (Fig. 3P) and quantification (Fig. 3Q) results revealed greater mineralization in LV-AMPKα2 than in LV-AMPKα1 cells on weeks 2.

**Figure 4:**
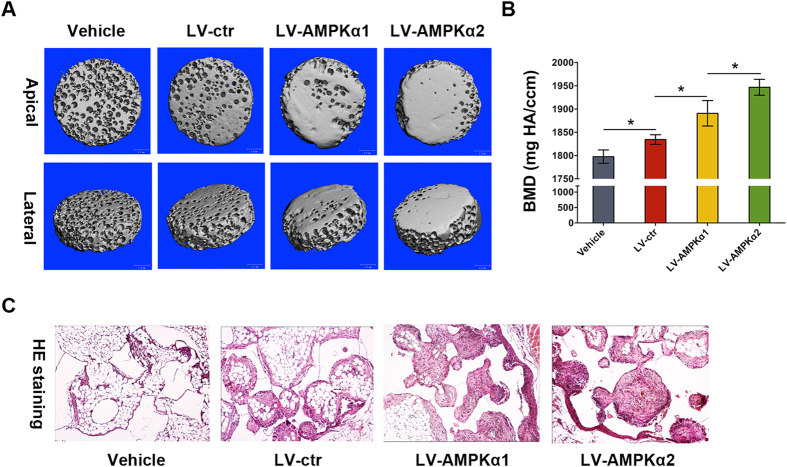
*In vivo* ectopic bone formation in MC3T3-E1 cells over-expressing AMPK α1 and α2. β-TCP scaffolds loading with and without MC3T3-E1 cells over-expressing AMPK α1 and α2 were implanted into the intramuscular pocket of the femur of nude mice. Eight weeks later, the complexes were harvested and scanned by micro-CT (**A**). Micro-CT images are shown in apical and lateral views. The measurement of BMD was performed on the basis of micro-CT (**B**). Then these complexes were decalcified, embedded in paraffin and stained with hematoxylin and eosin (H&E). Data were shown as the mean ± SD. *p < 0.05.

Further, we examined ectopic bone formation of LV-AMPKα1 and LV-AMPKα2 MC3T3-E1 cells in nude mice. We implanted β-TCP scaffolds loading with and without MC3T3-E1 cells into the intramuscular pocket of nude mice. Eight weeks after implantation, we harvested the specimens and subjected them to micro-CT imaging and hematoxylin and eosin (H&E) staining. LV-AMPKα MC3T3-E1 cells induced more ectopic bone formation compared to LV-ctr MC3T3-E1 cells (Fig. 4A,B), indicating that AMPK could enhance *in vivo* osteogenesis of MC3T3-E1 cells. Importantly, much more ectopic bone formation was observed in LV-AMPKα2 MC3T3-E1 cells compared with LV-AMPKα1 MC3T3-E1 cells (Fig. 4A,B). H&E staining revealed the same pattern as micro-CT (Fig. 4C).

### Osteoblast-associated osteoclastogenesis of bone marrow monocytes is attenuated by AMPK α2 compared with α1

**Figure 5:**
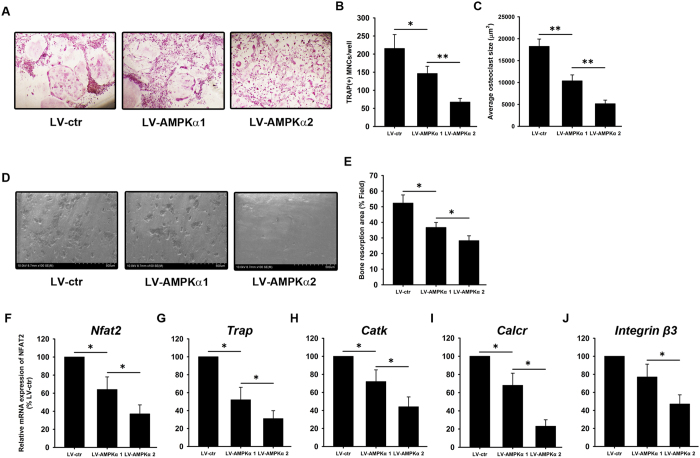
Osteoclastogenesis in bone marrow monocytes induced by AMPK α1- and α2-expressing MC3T3-E1 cells. Bone marrow monocytes were co-cultured with LV-AMPKα1 and LV-AMPKα2 MC3T3-E1 cells in the presence of BMP2. On day 7, cells were assessed by TRAP staining (**A**) followed by quantification of TRAP + monocytes (MNC) (**B**) and average osteoclast size (**C**). Osteoclast function was assessed by osteoclast cultures on bone slice followed by SEM imaging (**D**). Percentage of resorption area was measured (**E**). qRT-PCR was performed to evaluate the mRNA expression of *Nfat2* (**F**), *Trap* (**G**), *Catk* (**H**), *Calcr* (**I**), and *integrin β3* (**J**). β-actin was used as an internal control. Data are shown as mean ± SD. *P < 0.05, **P < 0.01.

Osteoclasts differentiation and maturation depends on RANKL and M-CSF secreted by osteoblasts, with the two cell types interacting through direct contact as well as paracrine signaling^14^. Here, the capacity for osteoclastogenic induction of MC3T3-E1 cells overexpressing the α1 or α2 subunits of AMPK was evaluated. Bone marrow monocytes were co-cultured with LV-AMPKα1 and LV-AMPKα2 MC3T3-E1 cells in the presence of BMP2, and tartrate-resistant acid phosphatase (TRAP) staining was carried out on day 7. Compared with LV-ctr, LV-AMPKα1 and LV-AMPKα2 MC3T3-E1 cells had weaker osteoclastogenic induction capacity, as indicated by TRAP staining (Fig. 5A) and evaluation of number (Fig. 5B) and average size (Fig. 5C) of TRAP^**+**^osteoclasts. More importantly, the number and average size of TRAP^**+**^osteoclasts were 60% lower and 40% smaller, respectively, in monocytes co-cultured with LV-AMPKα2 as compared to LV-AMPKα1 MC3T3-E1 cells (Fig. 5A–C).

Further, we investigated the osteoclast function induced by LV-AMPKα1 and LV-AMPKα2 MC3T3-E1 cells by evaluating resorption pit formation. The results revealed lower osteoclast function induced by LV-AMPKα1 and LV-AMPKα2 MC3T3-E1 cells compared with LV-ctr cells (Fig. 5D,E). Moreover, lower osteoclast function induced by LV-AMPKα2 cells was observed compared with LV-AMPKα1 cells (Fig. 5D,E).

qRT-PCR analysis revealed a downregulation in the expression of osteoclastogenic markers including nuclear factor of activated T cells 2 (*Nfat2*), *Trap*, cathepsin K (*Catk*), calcitonin receptor (*Calcr*), and *integrin β3* in bone marrow monocytes co-cultured with LV-AMPKα1 and LV-AMPKα2 cells compared with LV-ctr cells (Fig. 5F–J). Moreover, more significant downregulation in the expression of these osteoclastogenic markers was observed in bone marrow monocytes co-cultured with LV-AMPKα2 cells compared with LV-AMPKα1 cells (Fig. 5F–J).

### Bone metabolism markers expression in MC3T3-E1 cells overexpressing AMPK α1 and α2 subunits

**Figure 6:**
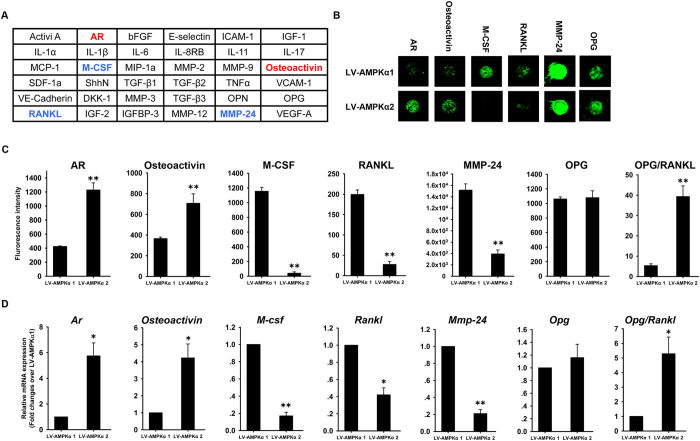
Secretion of proteins involved in bone metabolism from MC3T3-E1 cells expressing AMPK α1 and α2. The levels of 36 proteins were measured using a high throughout antibody array and are shown as upregulation (red) or downregulation (blue) in α2-AMPK vs. α1-AMPK MC3T3-E1 cells (**A**). Fluorescent signals (**B**) and fluorescence intensity (**C**) corresponding to AR, osteoactivin, M-CSF, RANKL, MMP24 and OPG were measured in MC3T3-E1 cells expressing α1- and α2-AMPK. The mRNA expression of AR, osteoactivin, M-CSF, RANKL, MMP24 and OPG in MC3T3-E1 cells expressing α1- and α2-AMPK was evaluated by qRT-PCR (**D**). β-actin was used as an internal control of qRT-PCR. Data are shown as mean ± SD. *P < 0.05, **P < 0.01.

Given that overexpressing the α2 as compared to the α1 subunit of AMPK conferred a greater osteogenic potential and diminished osteoblast-associated induction of osteoclastogenesis, the potential mechanisms underlying this difference were investigated in cells treated with BMP2 for 7 days by evaluating the protein expression levels of 36 markers of bone metabolism using an antibody array (Fig. 6A). The relative expression level of specific proteins was represented by the fluorescent signal intensity. Among the 36 markers, five markers that were differentially expressed between LV-AMPKα1 and LV-AMPKα2 MC3T3-E1 cells were of particular interest (Fig. 6A,B). AR and osteoactivin were upregulated by 180% and 110%, respectively, and M-CSF, RANKL, and matrix metalloproteinase (MMP)-24 were downregulated by >90%, 80%, and 70%, respectively, in LV-AMPKα2 as compared to LV-AMPKα1 cells (Fig. 6C). There was no significant difference in OPG expression level between LV-AMPKα1 and LV-AMPKα2 MC3T3-E1 cells, so that higher OPG/RANKL ratio was observed in LV-AMPKα2 cells as compared to LV-AMPKα1 cells (Fig. 6C). The expression profiles of the other markers are shown in Supplementary Figure 1. In LV-AMPKα1 and LV-AMPKα2 cells treated with BMP2 for 7 days, qRT-PCR analysis revealed an upregulation of *Ar* and *Osteoactivin* and downregulation of *M-csf, Rankl*, and *Mmp-24* mRNA levels in LV-AMPKα2 relative to LV-AMPKα1 cells (Fig. 6D), consistent with the protein expression data from the antibody array.

### Osteoactivin and AR are involved in osteogenesis of AMPK α subunit-overexpressing cells

**Figure 7:**
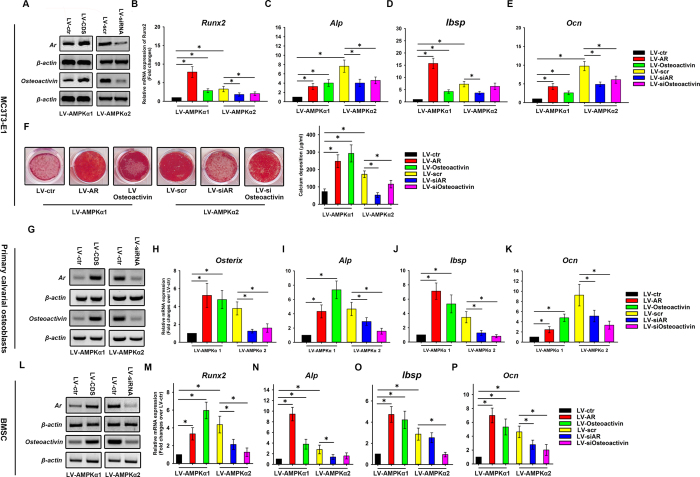
Modulation of osteogenesis in MC3T3-E1, primary osteoblasts and mouse BMSCs expressing α1 and α2 subunits of AMPK via AR and osteoactivin. AR and osteoactivin coding sequences (CDS) were expressed in α1-AMPK MC3T3-E1 (**A**), primary osteoblasts (**G**) and mouse BMSCs (**L**) and their expression was knocked down in α2-AMPK MC3T3-E1 (**A**), primary osteoblasts (**G**) and mouse BMSCs (**L**). An empty LV vector (LV-ctr) and a vector expressing scrambled siRNA (LV-scr) served as the respective controls. MC3T3-E1, primary osteoblasts and mouse BMSCs were induced to osteogenic differentiation. On day 7 (MC3T3-E1 and mouse BMSCs) and day 9 (primary osteoblasts) after induction, the expression of the osteogenesis markers *Runx2* (**B,M**), Osterix (**H**), *Alp* (**C,I,N**), *Ibsp* (**D,J,O**), and *Ocn* (**E,K,P**) was evaluated by qRT-PCR. β-actin was used as an internal control of qRT-PCR. On day 21 after induction, calcium deposition was assessed by alizarin red staining and quantification (**F**). Data are shown as mean ± SD; *P < 0.05.

Osteoactivin and AR play essential roles in osteogenic differentiation^15^,^16^,^17^,^18^. Since the expression of both proteins was higher in MC3T3-E1 cells overexpressing the α2 compared with the α1 subunit of AMPK, we investigated whether this was responsible for a greater osteogenic potential in LV-AMPKα2 as compared to LV-AMPKα1 cells. To answer this question, LV-AMPKα1 and LV-AMPKα2 MC3T3-E1 cells were infected, respectively, with LV particles expressing AR and osteoactivin (Fig. 7A) and siRNA against the two proteins (Fig. 7A). The cells were then induced to osteogenesis. A qRT-PCR analysis performed on day 7 showed that the mRNA expression of *Runx2, Alp, Bsp*, and *Ocn* was upregulated in response to AR and osteoactivin overexpression in LV-AMPKα1 cells (Fig. 7B–E). In contrast, AR knockdown resulted in the downregulation of *Runx2, Alp, Ibsp*, and *Ocn* mRNA expression in LV-AMPKα2 cells (Fig. 7B–E). Osteoactivin knockdown also decreased the mRNA expression of *Runx2, Alp*, and *Oc*n, but had no effect on the level of *Ibsp*. On day 21 after induction, Alizarin Red staining and quantification revealed greater mineralization in LV-AMPKα1 cells overexpressing AR and osteoactivin than in controls, whereas AR and osteoactivin knockdown reduced mineralization in LV-AMPKα2 cells (Fig. 7F).

We confirmed these results in LV-AMPKα1 and LV-AMPKα2 primary osteoblasts (Fig. 7G). A qRT-PCR analysis performed on day 9 showed that the mRNA expression of *Osterix, Alp, Ibsp*, and *Ocn* was upregulated in response to AR and osteoactivin overexpression in LV-AMPKα1 cells (Fig. 7H–K). In contrast, AR and osteoactivin knockdown resulted in the downregulation of *Osterix, Alp, Ibsp* and *Ocn* mRNA expression in LV-AMPKα2 cells (Fig. 7H–K).

We also confirmed the results described above in LV-AMPKα1 and LV-AMPKα2 BMSCs (Fig. 7L). A qRT-PCR analysis performed on day 7 showed that the mRNA expression of *Runx2, Alp, Ibsp*, and *Ocn* was upregulated in response to AR and osteoactivin overexpression in LV-AMPKα1 cells (Fig. 7M–P). In contrast, AR knockdown resulted in the downregulation of *Runx2, Alp*, and *Oc*n mRNA expression, but had no effect on the level of *Ibsp* in LV-AMPKα2 cells (Fig. 7M–P). Osteoactivin knockdown also decreased the mRNA expression *Runx2, Ibsp* and *Ocn*, but had no effect on the level of *Alp* (Fig. 7M–P).

### Adipogenic potential is suppressed by overexpression of the AMPK α2 subunit

**Figure 8:**
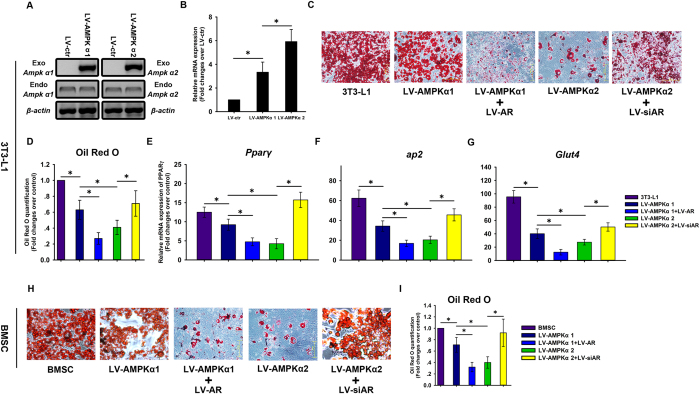
Adipogenesis in 3T3-L1 and mouse BMSCs expressing AMPK α1 and α2 subunits. LV vectors expressing AMPK α1 and α2 subunits were used to infect 3T3-L1 cells, and positive clones were selected with puromycin. An empty LV vector served a control (LV-ctr). The expression of exogenous and endogenous AMPK α1 and α2 was evaluated by RT-PCR (**A**). AR mRNA expression level was evaluated by qRT-PCR (**B**). β-actin was used an internal control. Adipogenesis was induced in infected cells in which AR was overexpressed (LV-AR) or knocked down (LV-siAR), and Oil Red O staining (**C**) and quantification (**D**) was used to assess the presence of fat droplets. The mRNA expression of the adipogenesis markers *Pparγ* (**E**), *aP2* (**F**), and *Glut4* (**G**) was evaluated by qRT-PCR. Adipogenesis was induced in mouse BMSCs overexpressing AMPK α1 and α2 in which AR was overexpressed (LV-AR) or knocked down (LV-siAR), and Oil Red O staining (**H**) and quantification (**I**) was used to assess the presence of fat droplets. Data are shown as mean ± SD. *P < 0.05.

The balance between osteogenesis and adipogenesis in BMSCs is impaired in several human diseases^19^, therefore its regulation is of medical importance^20^. The role of AMPK in adipogenesis was investigated in preadipocyte 3T3-L1 cells stably expressing AMPK α1 or α2 subunits. Exogenous AMPK α1 and α2 subunits were expressed at high levels in LV-AMPKα1 and LV-AMPKα2 3T3-L1 cells (Fig. 8A) respectively, as determined by RT-PCR. The expression of endogenous AMPK α1 or α2 was unaffected by the overexpression of the exogenous proteins (Fig. 8A).

LV-AMPKα1 and LV-AMPKα2 3T3-L1 cells were then compared in terms of AR expression. *Ar* mRNA level was upregulated in LV-AMPKα1 and LV-AMPKα2 cells compared with LV-ctr cells (Fig. 8B). Further, there was upregulation in *Ar* mRNA level in LV-AMPKα2 compared with LV-AMPKα1 cells (Fig. 8B). Oil Red O staining (Fig. 8C) and quantification (Fig. 8D) revealed that there were fewer intracellular fat droplets in LV-AMPKα1 and LV-AMPKα2 cells than in 3T3-L1 cells. More importantly, the formation of intracellular fat droplets was decreased to a greater degree by overexpressing AMPK α2 as compared to AMPK α1 (Fig. 8C,D). A qRT-PCR analysis showed that transcript expression levels of adipogenesis markers including peroxisome proliferator-activated receptor γ (*Pparγ*), *aP2*, and glucose transporter 4 (*Glut4*) were downregulated in LV-hAMPKα1 and LV-hAMPKα2 as compared to 3T3-L1 cells, with AMPKα2 having the greater effect (Fig. 8E–G).

AR level is negatively correlated with adipogenesis in BMSCs^18^. Given the upregulation in AR expression and inhibition of adipogenesis induced by AMPK α2 as compared to AMPK α1 overexpression, a role for AR in adipogenesis was examined by infecting LV-AMPKα1 and LV-AMPKα2 3T3-L1 cells with a virus expressing AR and a siRNA against AR respectively, and inducing adipogenic differentiation. On day 15, Oil Red O staining and quantification were used to evaluate adipogenesis and cells were analyzed for *Pparγ, aP2*, and *Glut4* expression by qRT-PCR. AR overexpression suppressed adipogenesis in LV-AMPKα1 3T3-L1 cells. The inhibition of adipogenesis was rescued in LV-AMPKα2 3T3-L1 cells by AR knockdown (Fig. 8C–G).

Further, we confirmed the results described above in LV-AMPKα1 and LV-AMPKα2 BMSCs. LV-AMPKα1 and LV-AMPKα2 BMSCs were infected with a virus expressing AR and a siRNA against AR respectively, and inducing adipogenic differentiation. On day 25, Oil Red O staining and quantification were used to evaluate adipogenesis. AR overexpression suppressed adipogenesis in LV-AMPKα1 BMSCs. The inhibition of adipogenesis was rescued in LV-AMPKα2 BMSCs by AR knockdown (Fig. 8H–I).

### Role of the AMPK α2 subunit in the impaired osteogenesis of BMSCs with FD

**Figure 9:**
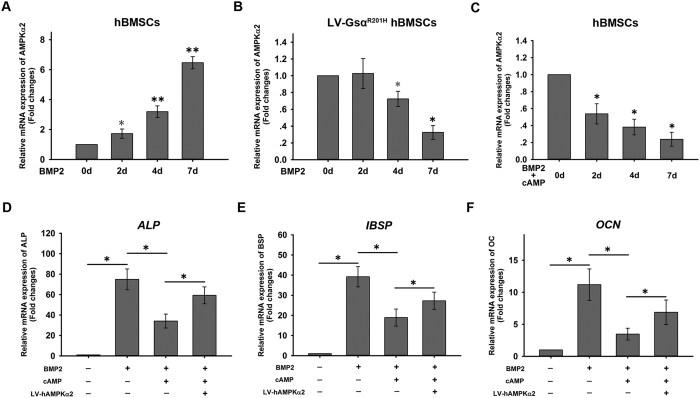
Role of AMPK α2 in the fibrous dysplasia (FD) phenotype. Two *in vitro* cell models were established that mimic the pathological features of FD: in one model, human BMSCs stably expressed a mutant GNAS protein harboring an R201H mutation (Gsα^R201H^); in the second model, human BMSCs were treated with an excess of membrane-permeable cAMP. Osteogenic differentiation was induced in the cells. On days 0, 2, 4, and 7, the expression of the AMPK α2 subunit was evaluated by qRT-PCR (**A–C**). Results are expressed as fold change in mRNA abundance relative to day 0 cultures. AMPK α2 subunit was overexpressed in cells with and without cAMP treatment. These cells were induced to osteogenesis. The mRNA expression of ALP (**D**), IBSP (**E**), and OCN (**F**) was evaluated by qRT-PCR. Data are shown as mean ± SD. *P < 0.05, **P < 0.01.

FD is characterized by an impairment in osteogenic differentiation potential in BMSCs due to activating missense mutations in the guanine nucleotide binding protein α-stimulating (*Gnas*) gene. In normal BMSCs, AMPK α2 mRNA expression was progressively upregulated during osteogenic differentiation (Fig. 9A). Two *in vitro* models were established that mimicked the pathological features of FD. In the first, BMSCs were generated that expressed a *Gnas* mutant harboring an R201H mutation (Gsα^R201H^); in the second model, BMSCs were treated with an excess of cell membrane-permeable cAMP^21^. Osteogenic differentiation was induced in the cells, and the mRNA expression of AMPK α2 was assessed on days 0, 2, 4, and 7. LV-Gsα^R201H^-BMSCs and excess cAMP-treated BMSCs showed the different pattern of AMPK α2 subunit mRNA expression with normal BMSCs: no statistical difference was observed at day 2 compared with day 0 and there was significant downregulation at day 4 (by 28%) and 7 (by 67%) compared with day 0 in LV-Gsα^R201H^-BMSCs (Fig. 9B). Accordingly, in excess cAMP-treated BMSC, there was gradual downregulation in AMPK α2 subunit mRNA expression at day 2 (by 46%), 4 (by 62%) and 7 (by 76%) compared with day 0 (Fig. 9C).

To determine whether the downregulation of the AMPK α2 subunit is responsible for the reduced osteogenic differentiation potential in BMSCs treated with excess cAMP, the AMPK α2 subunit was overexpressed by LV transduction in these cells, which were then induced to undergo osteogenic differentiation. Overexpression of the AMPK α2 subunit rescued the impaired osteogenic differentiation in cAMP-treated BMSCs, as evidenced by the increased *ALP, IBSP*, and *OCN* transcript levels (Fig. 9D–F), implicating the role of α2 subunit of AMPK in FD pathogenesis.

## Discussion

The results of the present study revealed three key functional differences between the AMPK α1 and α2 subunits. The α2 subunit increased osteogenic potential in MC3T3-E1 cells, primary osteoblasts and mouse BMSCs, which involved AR and osteoactivin. Compared to the α1 subunit of AMPK, the α2 subunit inhibited osteoblast-associated induction of osteoclastogenesis in MC3T3-E1 cells via downregulation of M-CSF and RANKL. Finally, compared to α1, overexpression of the α2 subunit suppressed adipogenesis in 3T3-L1 cells, which also involved AR.

Mammalian AMPK has two isoforms of the α and β and three of the γ subunit, and the various isoforms of each subunit have distinct biological functions^22^,^23^. A769622 activates AMPK both allosterically and by inhibiting the dephosphorylation of AMPK α^Thr172^ by specific phosphatases^24^. Its mode of action does not involve binding to the γ subunit, unlike other AMPK activators such as AMP and AICAR, but instead depends on the presence of the β subunit^25^. Interestingly, it was shown that A769622 is selective for the β1 isoform and does not activate AMPK heterotrimers containing β2 subunits^26^. In contrast, only AMPK β2 can be modified posttranslationally by PIASy-dependent SUMOylation, leading to activation of the AMPK complex^25^. One study screened 12,000 genes in the H1299 human lung carcinoma line and found 133 genes that were either induced or repressed in response to p53-dependent cell growth arrest and apoptotic conditions, including β1 but no other AMPK subunits^27^. Another study demonstrated that α1 and γ1 are almost exclusively localized in the cytoskeleton, while α2 and γ2 are present in all subcellular fractions, including the nucleus^28^. These data suggest that pharmacological interventions targeted to specific AMPK subunit isoforms can selectively modify particular AMPK functions.

The α subunit of AMPK is the main catalytic domain of the AMPK complex. A dominant-negative α2 subunit attenuated the mutant AMPK γ2 phenotype, and AMPK complexes containing α2 rather than α1 subunit mediate the effects of AMPK γ2 mutations^22^. AMPK α2 is the main effector of basal and AICAR-stimulated AMPK activity, including AICAR-induced glucose uptake^29^. Clot retraction was impaired in platelets from AMPKα2^−/−^ but not AMPKα1^−/−^ mice^23^, and AMPK α2 knockout mice showed increased sensitivity to diet-induced obesity and insulin resistance, whereas no metabolic defects were observed in α1 knockout mice.

Few reports have compared the functions of AMPK α1 and α2 subunits. The AMPK agonists AICAR and metformin induce osteogenesis in MC3T3-E1 cells^30^,^31^,^32^,^33^,^34^, and metformin causes increases in ALP activity, collagen synthesis, OC production, and extracellular Ca^2+^ deposition *in vitro*, possibly by increasing *Runx2* expression. When primary osteoblasts were co-treated with AICAR and the AMPK antagonist compound C, the latter suppressed the stimulatory effect of the agonist on bone nodule formation. The present study analyzed differences in the osteogenic potential of MC3T3-E1 cells expressing the α1 or α2 subunits of AMPK. The findings that the α2 subunit conferred the cells with greater osteogenic potential as compared to α1 are at variance with those of another study, which found no differences in tibial bone mass between AMPK α2 knockout and wild-type mice, although both cortical and trabecular bone compartments were smaller in the mutants^35^. In addition to possible differences attributable to the model systems that were used (*in vivo* vs. *in vitro* in this study), one possible reason for the discrepancy between the findings is the low expression of α2 relative to the α1 isoform in the skeletal system.

α1 is the predominant AMPK isoform expressed (albeit at low levels) in BMSCs, preosteoblasts, and preadipocytes^8^,^35^,^36^. The antibody array data presented here showed that the levels of many markers of bone metabolism differed between MC3T3-E1 cells expressing α1 or α2 subunits, including AR, basic fibroblast growth factor, interleuking-6 and -17, MCP-1, M-CSF, macrophage inflammatory protein 1a, MMP9, osteoactivin, tumor necrosis factor α, RANKL, and MMP24 (Supplementary Fig. 1). Of these, osteoactivin and especially AR were implicated in the differences in osteogenesis potential between α1 and α2. This is consistent with studies showing that AR deficiency leads to tissue-nonspecific ALP downregulation followed by decreased phosphate production, ultimately reducing bone mineralization. Similar results were observed in osteoblast-specific AR knockout mice, in which AR was found to stimulate osteoblast differentiation and suppress bone resorption^37^,^38^; AR mutants developed osteoporosis and showed decreased BMSC osteogenesis resulting from the downregulation of *Runx2*^39^.

In addition, an enhancement of osteogenesis, AR also suppresses adipogenesis. Androgen treatment inhibits adipocyte differentiation and body fat formation *in vitro* and in rodent and nonhuman primate models, as well as rosiglitazone-induced adipogenesis in human BMSCs; it also promotes interactions between β-catenin, AR, and T-cell factor 4 to suppress adipogenic differentiation of 3T3-L1 cells^40^, which were induced to undergo adipogenesis by overexpression of the α1 and α2 subunits of AMPK in this study. The weaker induction in α2-AMPK 3T3-L1 corresponded to a greater upregulation in AR expression as compared to the adipogenic induction and AR level in α1-AMPK 3T3-L1 cells. Adipogenesis was rescued in α2-AMPK 3T3-L1 and inhibited in α1-AMPK 3T3-L1 cells upon AR knockdown and overexpression, respectively, suggesting that the functional differences between α1 and α2 involve AR signaling.

Osteoclast differentiation and maturation depend on cues from the microenvironment, especially from osteoblasts through cell-cell contact and paracrine signaling. This study provides evidence that osteoblast-associated osteoclastogenesis was reduced in bone marrow monocytes co-cultured with MC3T3-E1 cells expressing α2 as compared to α1 and likely involves RANKL and M-CSF, as the expression of these two proteins was downregulated in the former. AR, which was upregulated in AMPK α2- relative to α1-expressing MC3T3-E1, may also be involved, since it inhibits bone resorption^41^ and AR-deficient mice exhibit a calvarial and femoral bone loss phenotype.

The most intriguing finding of the present study as that AMPK α2 expression was impaired during osteogenesis in an FD model. In normal BMSCs, α2 subunit transcript level gradually increased during osteogenic differentiation; however, this was abrogated and even reversed in BMSCs exhibiting the FD phenotype, and the defect in osteogenesis in these cells was rescued by overexpression of AMPK α2, suggesting that the α2 subunit may be a critical factor in the pathogenesis of FD. For instance, α2 may be involved in the overactivation of bone resorption as our findings demonstrate a negative relationship between α2 expression and osteoclastogenesis.

In conclusion, the results from this study indicate that the α2 and α1 subunits of AMPK have several functional differences, with α2 conferring stronger osteogenic potential and a weaker ability to induce osteoblasts-associated osteoclastogenesis in MC3T3-E1 cells as well as conferring a lower adipogenic potential to 3T3-L1 cells. These findings provide a basis for developing drugs that can differentially target the α1 and α2 subunits of AMPK to treat diseases such as obesity and osteoporosis that are associated with mutations in specific AMPK subunits.

## Materials and Methods

### Cell cultures

#### MC3T3-E1 cells

MC3T3-E1 cells were cultured in α-minimal essential medium (α-MEM) (Gibco, Grand Island, NY, USA) supplemented with 10% fetal bovine serum (FBS), 100 U/ml penicillin, and 100 mg/ml streptomycin (all from Hyclone, Logan, UT, USA). In all experiments, cells were cultured in a humidified atmosphere at 37 °C and 5% CO_2_ with the medium changed every 3 days.

MC3T3-E1 cells were treated with recombinant human bone morphogenetic protein (BMP)2 (R&D, Minneapolis, MN, USA) at a final concentration of 100 ng/ml to induce osteogenesis.

#### Primary calvarial osteoblasts

Four-day-old male C57BL/6J mice were killed with a lethal dose of sodium pentobarbital. Calvaria was dissected, cleaned of soft tissue, and maintained in PBS buffer. The cleaned calvaria was cut into small pieces of 1–2 mm^2^ and washed with PBS. The bone pieces were incubated with 2 mg/ml collagenase II (Sigma, St. Louis, MO) solution for 2 h at 37 °C in a shaking water bath. Then, the fragments were washed with α-MEM supplemented with 10% FBS and cultured in culture medium (α-MEM containing 10% FBS, 100 U/ml penicillin, 100 μg/ml streptomycin). The culture flasks were stored in a humidified atmosphere of 5% CO_2_ in air at 37 °C. After confluence, the bone fragments were removed and the confluent layers were trypsinized and the cells were replated.

Primary calvarial osteoblasts were induced to osteogenesis by Osteogenesis Induction Medium (OIM) containing 10 mM β-glycerophosphate and 100 μg/ml ascorbic acid.

#### BMSCs

Mouse BMSCs were purchased from Cyagen Bioscience Inc. Human BMSCs were isolated and expanded using a modified version of a previously described method^42^,^43^. The donor was healthy and had no metabolic or other diseases or inherited conditions that could affect the current study. Written informed consent was obtained from the donor, and the study was approved by the Ethics Committee of Shanghai Ninth People’s Hospital Affiliated to Shanghai JiaoTong University School of Medicine. Methods used in this study were carried out in accordance with the the relevant guidelines and regulations.

Mouse BMSCs were induced to osteogenesis by Osteogenesis Induction Medium (OIM) containing 10 mM β-glycerophosphate, 100 μg/ml ascorbic acid, and 10 nM dexamethasone. To investigate the effect of excess cAMP on AMPK α1 and α2 expression in human BMSCs, confluent cells were exposed to medium containing BMP2 and 2 mM dibutyryl-cAMP (Sigma, St. Louis, MO, USA).

#### 3T3-L1 cells

3T3-L1 cells were cultured in Dulbecco’s Modified Eagle’s Medium (DMEM) (Gibco) supplemented with 10% FBS, 100 U/ml penicillin, and 100 mg/ml streptomycin. Adipogenic differentiation of 3T3-L1 and mouse BMSCs was induced as previously described^20^. Briefly, confluent cells were treated with a complete adipogenic hormone cocktail consisting of DMEM supplemented with 10% FBS, 10 g/ml insulin, 0.5 mM methylisobutylxanthine, and 1 μM dexamethasone (all from Sigma). The day differentiation was induced was considered as day 0. On day 3, the culture medium was replaced with DMEM containing only insulin and 10% FBS. On day 6, the complete adipogenic hormone cocktail was again added.

### Osteoblast-associated osteoclastogenesis

Bone marrow monocytes isolated from long bones of 6-week-old C57BL/6 mice were cultured with MC3T3-E1 in the presence of BMP2 (100 ng/ml) for 7 days. The cells were then fixed and stained for TRAP. TRAP-positive multinuclear cells (>3 nuclei/cell) were considered as osteoclasts and were counted. The total area of TRAP-positive regions were quantified using Image J software (National Institutes of Health, Bethesda, MD, USA) in each sample in 5 randomly selected fields of view.

### Bone absorption assay

Bone marrow monocytes were cultured with MC3T3-E1 in the presence of BMP2 (100 ng/ml) for 7 days on bovine bone slices. Cells on bone slices were then removed by mechanical agitation and sonication. Bone resorption pits were visualized under a scanning electron microscope (SEM) (FEI Quanta 250), and the percentage of bone resorption area was quantified using Image J software.

### Reverse transcription (RT)- and real-time PCR

Total cellular RNA was isolated from cultured cells using the RNeasy Mini Kit (Qiagen, Valencia, CA, USA) according to the manufacturer’s protocol. Primers used in all reactions are shown in Supplementary Table 1. For RT-PCR, single-stranded cDNA was reverse-transcribed from 1 μg total RNA using reverse transcriptase with an oligo-dT primer. PCR was carried out with 1 μl cDNA using the following cycling parameters: 30 cycles of 94 °C for 40 s, 60 °C for 40 s, and 72 °C for 40 s. PCR products were analyzed by agarose gel electrophoresis. Real-time PCR was carried out on a 96-well plate ABI Prism 7500 Sequence Detection system (Applied BioSystems, Foster City, CA, USA) using SYBR Green PCR Master Mix (Takara Bio Inc., Otsu, Japan). Cycling conditions were as follows: 40 cycles of 94 °C for 5 s, 60 °C for 34 s, and 72 °C for 30 s. The comparative 2^−ΔΔCt^ method was used to calculate the relative expression of each target gene as previously described^42^; the expression levels of all genes were normalized to that of β-actin.

### Lentiviral transduction

LV vectors containing the coding sequences of human AMPK α1 and α2 subunits, AR, and osteoactivin and short interfering (si)RNAs against AR and osteoactivin were purchased from Genecopoeia (Rockville, MD, USA). LV particles were generated as previously described^21^. Briefly, 1.3–1.5 × 10^6^ 293T cells were plated in a 10-cm dish and the transfection complex was added directly to the culture medium when cells reached 70–80% confluence. After incubation in a CO_2_ incubator at 37 °C, LV particle-containing medium was collected 48 h post-transfection.

### Bone metabolism markers array

Semi-quantitative sandwich-based bone metabolism markers arrays (Human Cytokine Array L-Series; RayBiotech, Atlanta, GA, USA) were used to detect 36 markers of bone metabolism on a glass slide matrix. Biotin-conjugated antibodies used for detection were combined as a single cocktail for later use. Printed slides were placed in chambers to allow incubation of each array with a different sample. Arrays were incubated in blocking buffer followed by whole cell lysis samples. After extensive washing to remove non-specific binding, the antibody cocktail was added to the arrays. After additional washes, the arrays were incubated with the streptavidin-conjugated HiLyte Fluor 532 (Anaspec, Fremont, CA), and the fluorescent signal was visualized using a GenePix 4200A laser-based scanner system (Molecular Dynamics, Sunnyvale, CA) on the green channel. Two replicates were spotted for each antibody, and the average median signal intensity for both spots (with the local background subtracted) was used to calculate protein level.

### Oil Red O staining and quantification

Cultured cells were stained with Oil Red O as previously described^19^ to evaluate adipogenesis. Briefly, cells were washed twice with phosphate-buffered saline (PBS) and fixed with 4% paraformaldehyde for 2 h at 4 °C. After two washes in PBS, cells were stained for 2 h in freshly diluted Oil Red O solution consisting of six parts Oil Red O stock solution (0.5% in isopropanol) and four parts water at 4 °C. The stain was removed from the cells with two PBS washes and cells were examined with an inverted microscope. To measure the quantification of lipid accumulation, Oil red O was eluted by adding 100% isopropanol and optical density was detected using a spectrophotometer at 520 nm.

### Alizarin Red staining and quantification

Osteogenesis was examined by staining mineralized nodules with Alizarin Red S. After fixation, cells were washed with PBS and soaked in 40 mM Alizarin Red (pH 4.2) for 30 min at 37 °C, then washed with PBS and imaged. Decalcification was performed using 0.1 M HCl overnight at 4 °C. Then, 20 μL of samples were transferred to the test tubes containing 1 mL of methyl thymol blue solution and 1 mL of alkaline solution. Absorbance was determined at 610 nm.

### Ectopic Bone Formation

All animal procedures were approved by the Animal Ethics Committee of Shanghai Ninth People’s Hospital Affiliated to Shanghai JiaoTong University School of Medicine and were performed in strict accordance with the NIH guidelines for the care and use of laboratory animals (NIH Publication No. 85e23 Rev. 1985). There were 4 groups in the experiment, (1) β-tricalcium phosphate (β-TCP) alone (Vehicle), (2) β-TCP loading LV-ctr MC3T3-E1 cells (LV-ctr), (3) β-TCP loading LV-AMPKα1 MC3T3-E1 cells (LV-AMPKα1) and (4) β-TCP loading LV-AMPKα2 MC3T3-E1 cells (LV-AMPKα2). A modified version of a previously described method was used^44^,^45^. All MC3T3-E1 cells were treated with rhBMP-2 at a final concentration of 100 ng/ml to induce osteogenesis for 3 days. Then approximately 2.0 × 10^6^ cells were seeded on each β-TCP disk (φ 6 mm × H 2 mm, Bio-lu Biomaterials Company, Shanghai, China). After 12 hours, the cell-scaffold complex was implanted into the intramuscular pocket of the femur of 8-week-old nude mice (BALB/c, nu/nu; SIPPR-BK Laboratory Animal Co. Ltd, Shanghai, China). Eight weeks after implantation, samples were harvested, fixed in 4% paraformaldehyde for micro-CT. Then these complexes were decalcified, and embedded in paraffin. Thin sections (5 μm) were stained with hematoxylin and eosin (H&E).

### Statistical analysis

Mean differences were evaluated by the Student’s t test for two-sample comparisons and one-way analysis of variance (ANOVA) for multiple comparisons using SPSS 16.0 (SPSS Inc., Chicago, IL, USA). Tukey’s test was used to identify significant differences in ANOVA. Data from at least three independent experiments were analyzed and are presented as the mean ± SD. P < 0.05 was defined as statistically significant.

## Additional Information

**How to cite this article**: Wang, Y.-g. *et al*. Functional differences between AMPK α1 and α2 subunits in osteogenesis, osteoblast-associated induction of osteoclastogenesis, and adipogenesis. *Sci. Rep.* **6**, 32771; doi: 10.1038/srep32771 (2016).

## Supplementary Material

Supplementary Information
