# Supplementary material for: Functional differences between AMPK α1 and α2 subunits in osteogenesis, osteoblast-associated induction of osteoclastogenesis, and adipogenesis
Source: Sci Rep. 2016 Sep 7;6:32771. doi: 10.1038/srep32771 (PMC5013406; doi:10.1038/srep32771)
Supplement: Supplementary Information [file srep32771-s1.pdf]

Functional differences between AMPK α1 and α2 subunits in osteogenesis, osteoblast-associated induction of osteoclastogenesis, and adipogenesis

Yu-gang Wang[1], Xiu-guo Han[1], Ying Yang[1], Han Qiao[1], Ke-rong Dai[1], Qi-ming Fan[1,]*, Ting-ting Tang[1,]*

[1]Shanghai Key Laboratory of Orthopedic Implants, Department of Orthopedic Surgery, Shanghai Ninth People's Hospital, Shanghai JiaoTong University School of Medicine, 639 Zhizaoju Road, Shanghai 200011, People's Republic of China

*** Corresponding author:**

Ting-ting Tang

Tel/fax: +8621 6313 7020

E-mail: tingtingtang@hotmail.com

Qi-ming Fan

Tel/fax: +8621 6313 9920

E-mail: chillow@163.com

Supplementary table 1: primer sequences

Gene	Primer sequences (5’-3’)	Product length (bp)
hAMPKα1	TTGAAACCTGAAAATGTCCTGCT (F)	113
	GGTGAGCCACAACTTGTTCTT (R)	
hAMPKα2	GTGAAGATCGGACACTACGTG (F)	100
	CTGCCACTTTATGGCCTGTTA (R)	
hALP	AACATCAGGGACATTGACGTG (F)	159
	GTATCTCGGTTTGAAGCTCTTCC (R)	
hBSP	CACTGGAGCCAATGCAGAAGA (F)	106
	TGGTGGGGTTGTAGGTTCAAA (R)	
hOC	GAGCCCCAGTCCCCTACCCG (F)	137
	GACACCCTAGACCGGGCCGT (R)	
hβ-actin	CATGTACGTTGCTATCCAGGC (F)	250
	CTCCTTAATGTCACGCACGAT (R)	
mRunx2	TTCAACGATCTGAGATTTGTGGG (F)	221
	GGATGAGGAATGCGCCCTA (R)	
mALP	CCAACTCTTTTGTGCCAGAGA (F)	110
	GGCTACATTGGTGTTGAGCTTTT (R)	
mBSP	ATGGAGACGGCGATAGTTCC (F)	148
	CTAGCTGTTACACCCGAGAGT (R)	
mOC	CTGACCTCACAGATCCCAAGC (F)	187
	TGGTCTGATAGCTCGTCACAAG (R)	
mAMPKα1	TACTCAACCGGCAGAAGATTCG (F)	220
	AGACGGCGGCTTTCCTTTT (R)	
mAMPKα2	CAGGCCATAAAGTGGCAGTTA (F)	156
	AAAAGTCTGTCGGAGTGCTGA (R)	
mNFAT2	TTCGAGTTCGATCAGAGCGG (F)	419
	TCTCTGTAGGCTTCCAGGCT (R)	
mTRACP	GAGAAAGTCAAGGGAGTGGCA (F)	330
	ATGATGAAGTCAGCGCCCAT (R)	
mCATK	GCCACGCTTCCTATCCGAAA (F)	483
	AGAGCTGAAAGCCCAACAGG (R)	
mCALCR	GCAGGCACTGCTAAGGAGA (F)	321
	TCCGCCTTTTCACTCTGGAC (R)	
mIntegrin β3	CTCCCTTCTTCCCTCCCCTC (F)	346
	GCCTCACTGACTGGGAACTC (R)	
mAR	TGAGTACCGCATGCACAAGT (F)	366
	TCAGGAAAGTCCACGCTCAC (R)	
mOsteoactivin	GTCCTGATCTCCATCGGCTG (F)	302
	AACAACAGTTCCCAGCCACA (R)	
mM-CSF	AAAAGCCACTCTTGGGGCAT (F)	414
	TGGTGAGGGGTCATAGAATCC (R)	
mRANKL	CCGAGACTACGGCAAGTACC (F)	446
	CCACATCCAACCATGAGCCT (R)	

Gene	Primer sequences (5'-3')	Product length (bp)
mMMP24	CACCGGTGTGTTGGATCAGA (F)	367
	ATGGGCTAGGAATCCCCCTT (R)	
mPhospho1	ATGAGCGGGTGTTTTCCAG (F)	102
	ATCGAAGTCGAAGGTGAGGAG (R)	
mOPG	CCTTGCCCTGACCACTCTTAT (F)	127
	CACACACTCGGTTGTGGGT (R)	
mβ-actin	GGCTGTATTCCCCTCCATCG (F)	154
	CCAGTTGGTAACAATGCCATGT (R)	

h: human, m: mouse, F: forward, R: reverse

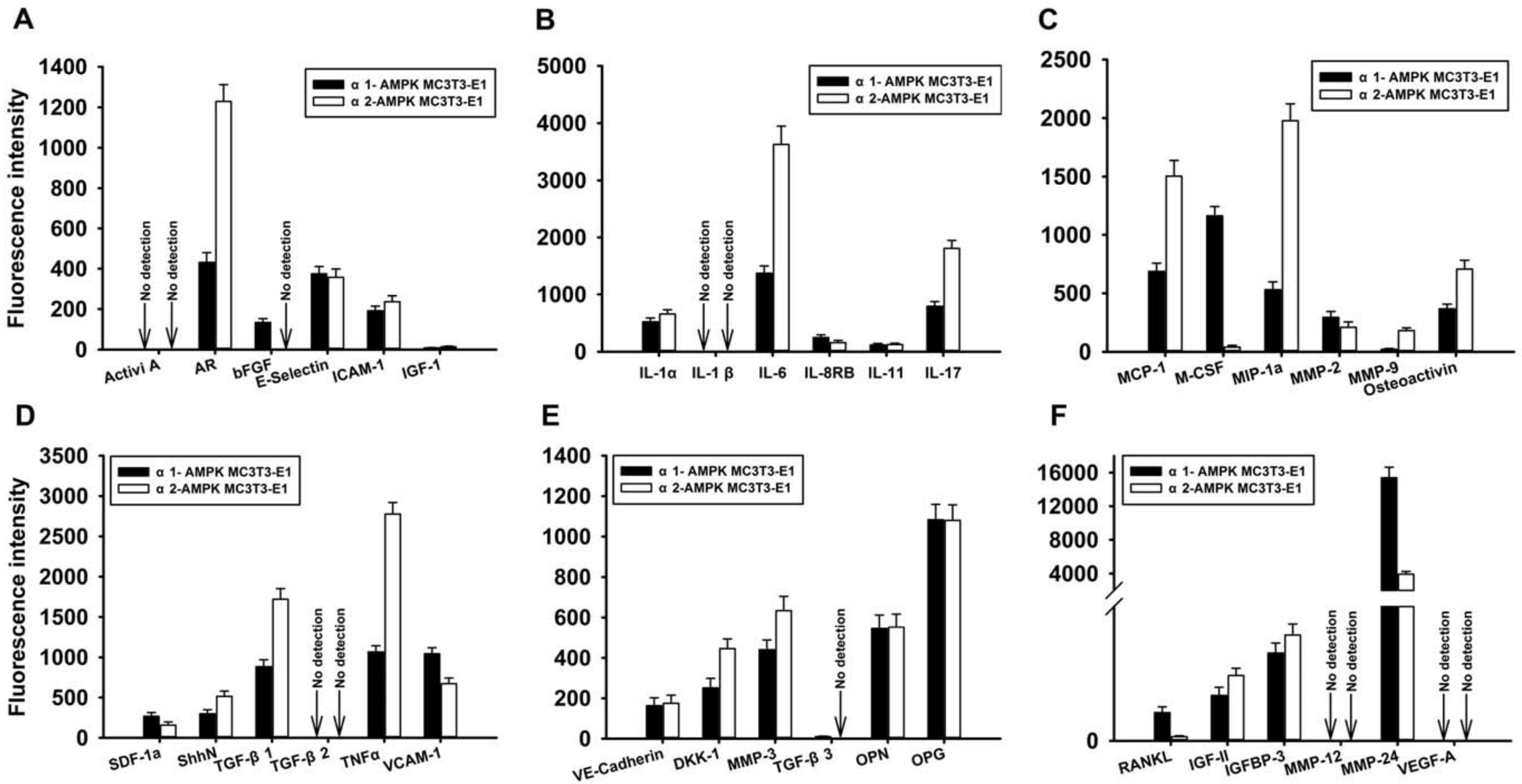

Supplementary figure 1

Supplementary figure 1: MC3T3-E1 cells infected with LV-hAMPKα1 or LV-hAMPKα2 were treated with BMP2 for 7 days. The protein expression of 36 markers of bone metabolisms was evaluated using an antibody array, with the relative expression level represented by fluorescent signal intensity.

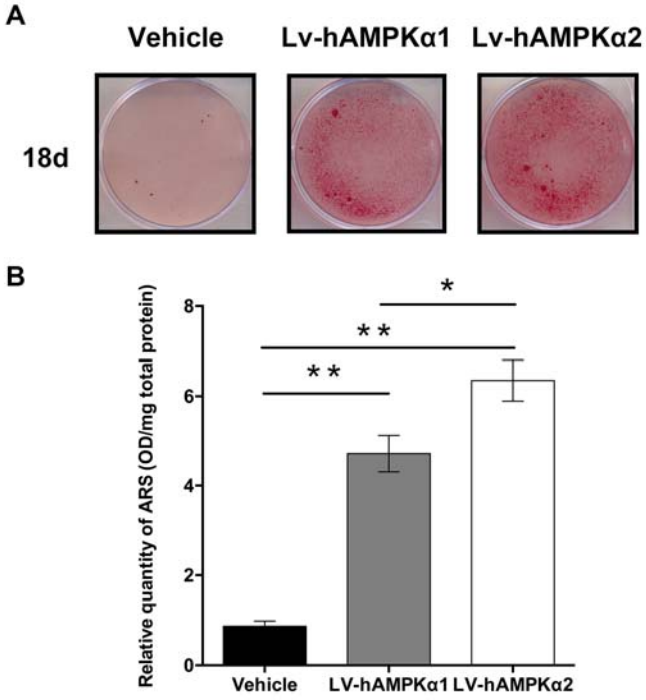

Supplementary figure 2

Supplementary figure 2. Osteogenesis in MC3T3-E1 cells expressing AMPK α1 and α2. Osteogenesis was induced with an osteogenic medium (100nM dexamethasone, 50μg/mL ascorbic acid and 10mM sodium β-glycerol-phosphate) for 18 days. **(A)** Calcium deposition was assessed by Alizarin Red staining. **(B)** Calcium deposition activity was assayed using colorimetrically quantitative analysis at 590 nm. Data are shown as mean ± SD. *P < 0.05, **P < 0.01.
